# Rice defense responses are induced upon leaf rolling by an insect herbivore

**DOI:** 10.1186/s12870-019-2116-0

**Published:** 2019-11-25

**Authors:** Jin-Hua Shi, Ze Sun, Xin-Jun Hu, Huanan Jin, Caroline Ngichop Foba, Hao Liu, Chao Wang, Le Liu, Feng-Feng Li, Man-Qun Wang

**Affiliations:** College of Plant Science and Technology, Huazhong Agricultural University, Wuhan, 430070 People’s Republic of China

**Keywords:** *Cnaphalocrocis medinalis*, Jasmonic acid, Salicylic acid, Volatiles, Transcriptome, Early detection

## Abstract

**Background:**

Plant defense against herbivores begins with perception. The earlier plant detects the harm, the greater plant will benefit in its arm race with the herbivore. Before feeding, the larvae of the rice pest *Cnaphalocrocis medinalis*, initially spin silk and fold up a leaf. Rice can detect and protect itself against *C. medinalis* feeding. However, whether rice could perceive *C. medinalis* leaf rolling behavior is currently unknown. Here, we evaluated the role of leaf rolling by *C. medinalis* and artificial leaf rolling in rice plant defense and its indirect effect on two important *C. medinalis* parasitoids (*Itoplectis naranyae* and *Apanteles* sp*.*) through a combination of volatile profiling, gene-transcriptional and phytohormonal profiling.

**Results:**

Natural leaf rolling by *C. medinalis* resulted in an increased attraction of *I. naranyae* when compared to the undamaged plant after 12 h. Volatile analysis revealed that six out of a total 22 components significantly increased in the headspace of *C. medinalis* rolled plant when compared to undamaged plant. Principal component analysis of these components revealed similarities in the headspace of undamaged plant and artificially rolled plant while the headspace volatiles of *C. medinalis* rolled plant deferred significantly. Leaf rolling and feeding by *C. medinalis* up-regulated the plant transcriptome and a series of jasmonic acid (JA) and salicylic acid (SA) related genes. While feeding significantly increased JA level after 12 to 36 h, rolling significantly increased SA level after 2 to 12 h. Compared to artificial rolling, natural rolling significantly increased JA level after 36 h and SA level after 2 and 12 h.

**Conclusions:**

Our findings suggest that natural leaf rolling by *C. medinalis* can be perceived by rice plant. The detection of this behavior may serve as an early warning signal in favor of the rice plant defenses against *C. medinalis*.

## Background

Throughout their life cycle, plants experience biotic and abiotic stresses, which can severely affect their growth and yield [1–3]. Globally, herbivore insects are among the most serious biotic problems and have close relationships with plants [4]. While insects have evolved ways to find hosts, plants are under selection pressure to evade detection or defend themselves when attacked [4–6].

To fend off insect herbivores, plants have evolved intricate and dynamic defense systems. Phytohormones play an important role in regulating plant defenses [7–9]. The phytohormones, jasmonic acid (JA) and salicylic acid (SA), and their derivatives play predominant roles in signal transduction of plant defenses against pathogens and herbivorous insects [10, 11]. Plant hormones can mediate downstream regulation of plant defenses, including direct defense mechanisms such as the production of defensive proteins and enzymes, and indirect defense mechanisms involving the production of volatile blends [12–14].

Plant volatiles are released naturally, and their emission can change in response to different stress factors [15, 16]. With plant–insect co-evolution, herbivore-induced plant volatiles (HIPVs) have become cost-saving weapons for plant defenses, and are useful in attracting natural enemies of herbivores [17–19]. Host recognition by natural enemies is specific, as they identify volatile blends from various plant species. However, natural enemies differ in their ability to distinguish HIPVs source depending on the distance of the natural enemy to the cues [20–22].

Conflicts in interactions of plants with herbivores begin with perception. Interestingly, plants can identify attacks from different insect species and mount several defenses [23, 24]. Plants perceive insect herbivores’ activities like oviposition, feeding, walking on the leaf surface, as well as chemical cues from insect oral secretion and frass [25–27]. Spinning is an important activity of silk-producing insects. The ability to produce silk has evolved in many groups of insects, and is used to accomplish a wide array of activities that enhance survival [28–30]. Several insects’ silk has been reported to attract parasitic wasps. For instance, *Apanteles melanoscelus* (Ratzeburg) (Hymenoptera: Braconidae) has a positive response to gypsy moth silk, and silk extracts of *Plodia interpunctella* (Hübner) (Lepidoptera: Pyralidae) larvae also attract parasitic wasps [31, 32]. However, no research has shown whether rolling behaviors following spinning can mediate plant defenses.

In Asia, the rice leaf folder, *Cnaphalocrocis medinalis* Guenée (Lepidoptera: Pyralidae), is one of the most important insect pest of paddy rice [33]. Severe feeding by this pest often affects the growth of rice plant leading to yield loss [34, 35]. Various wasp parasitoids of *C. medinalis* commonly exist in rice fields, such as *Itoplectis naranyae* (Ashmead) (Hymenoptera: Ichneumonidae) and *Apanteles* sp*.* (Hymenoptera: Braconidae) [36, 37]. The characteristic behavior of *C. medinalis* larvae is to roll a leaf longitudinally, by spinning silk before feeding [38, 39]. There are reports indicating that the feeding behavior of *C. medinalis* can induce rice defenses, but there is no evidence to indicate whether leaf rolling alone prior to feeding by the pest, can be perceived by the plant [40, 41].

Early detection of herbivores is beneficial for plants to develop effective and sustainable defenses. It is therefore important to determine whether rice plant can detect the early threat of *C. medinalis* leaf rolling, by initiating defenses against the pest [42]. In this study, we investigated the dynamics of rice plant responses to *C. medinalis* leaf rolling, *C. medinalis* leaf rolling and feeding and artificial leaf rolling, and compared with undamaged plant. To achieve this goal, we integrated results from wasps’ behavioral assays, plant volatile profiles, plant transcriptional and phytohormonal profiles.

## Results

### Behavior of natural enemies

**Fig. 1:**
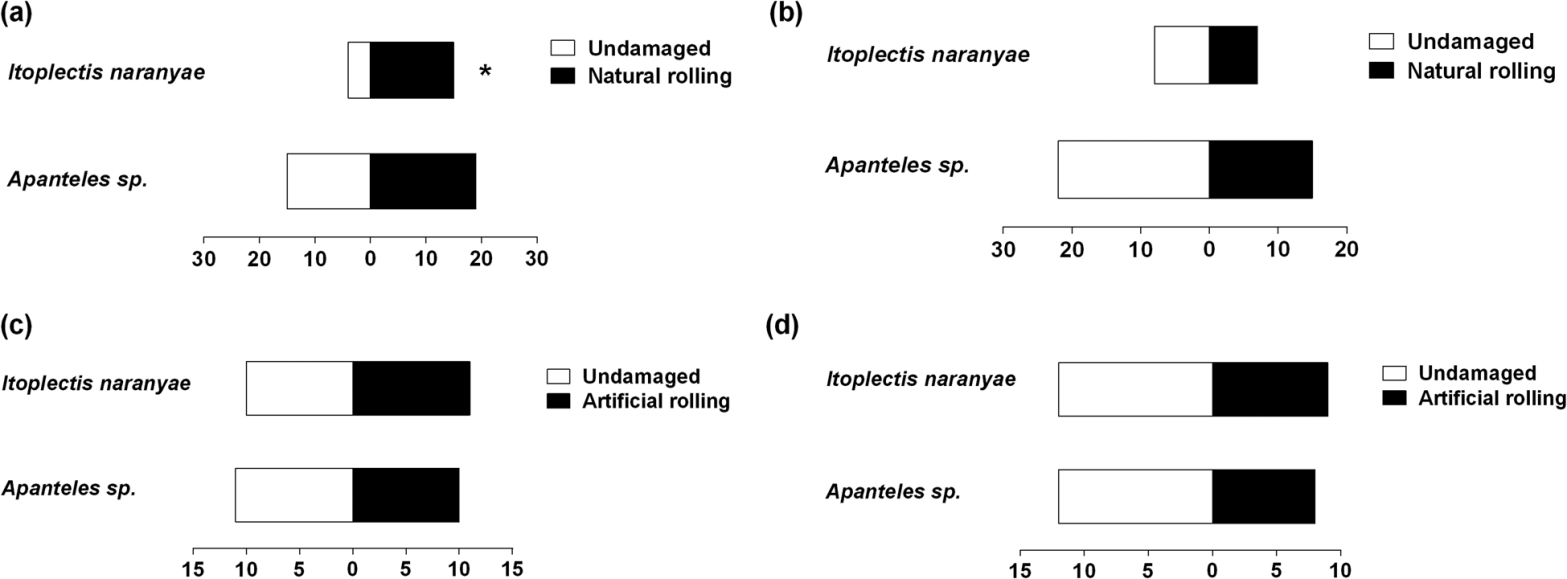
Olfactometer responses of *Itoplectis naranyae* and *Apanteles* sp*.* to odors f from artificial rolling plant and natural rolling plant treatments after 12 h (**a, c**) and 24 h (**b, d**) versus undamaged plant. Each wasp was released one at a time in the main arm of the Y-tube and given 5 min to make a choice between the test or control odor. Means with asterisk are significantly different (*P* < 0.05)

We hypothesized that leaf rolling behavior of *C. medinalis* larvae can trigger rice plant defenses. Therefore, we tested the preferences of two parasitic wasps (*I. naranyae* and *Apanteles* sp*.*) associated with *C. medinalis* larva. *Itoplectis naranyae* significantly preferred natural rolling treatment, when compared to undamaged plant after 12 h (*x*^*2*^ = 6.3684, *df* = 1, *P* = 0.0116) (Fig. 1a). However, both wasp species showed no significant preferences for naturally rolled plant after 24 h induction, when compared to undamaged plant (Fig. 1b). To explore whether sole rolling of plant leaf could be detected by natural enemies, we also tested the behavior choices of wasps between undamaged plant and artificially rolled plant. However, no differences in preferences for both wasp species between undamaged plant and artificially rolled plant were observed after 12 and 24 h (Fig. 1c and d).

### Analysis of rice plant volatiles

**Fig. 2:**
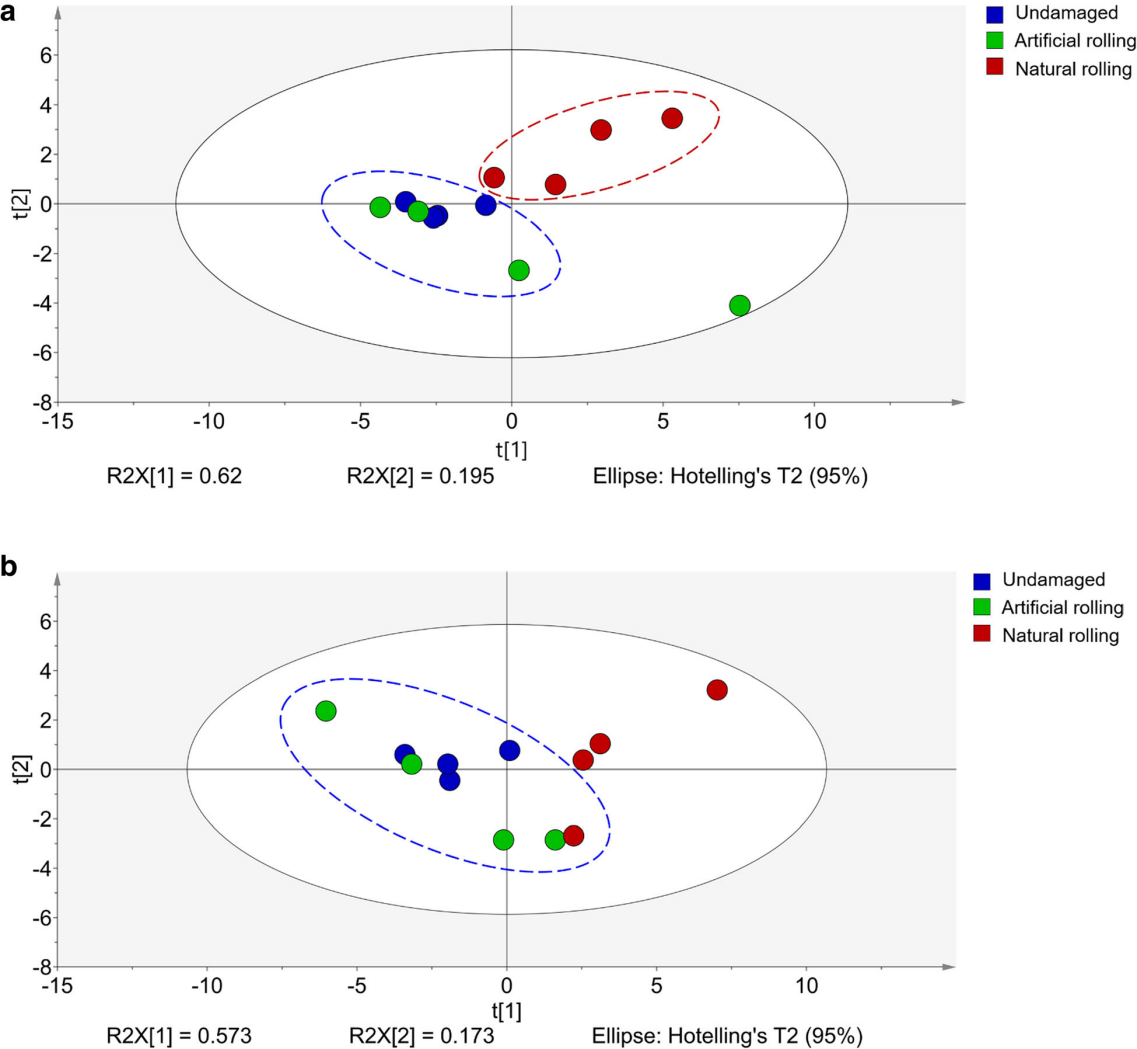
Principal Component Analysis comparing volatile blends from undamaged plant, artificial rolling and natural rolling plant treatments. **a** after 12 h attack and (**b**) after 24 h attack. Grouping pattern of samples were with respect to the first two principal components and Hotelling’s ellipse at 95% confidence interval for the observations. Each point represents one sample

To explore the reason for natural enemy behavior, we analyzed plant volatiles in undamaged plant, artificially rolled plant, and naturally rolled plant. In total, 22 volatile components were identified, including several terpenes, aldehydes, ketones, and esters (Table 1). Generally, these components occurred at lower amounts in the headspace of undamaged plant and artificially rolled plant, and at higher amounts in the headspace of naturally rolled plant. Of the 22 components identified, six were significantly higher after 12 and 24 h in the headspace of naturally rolled plant (*P <* 0.05). Principal Component Analysis also showed that the headspace volatiles of undamaged plant and artificially rolled plant were similar, but the headspace volatiles of naturally rolled plant differed significantly from both undamaged plant and artificially rolled plant (*P <* 0.05), especially after 12 h of damage (Fig. 2a and b).

**Table 1:** The amounts of volatile compounds (mean % of internal standard ± SEM) emitted from undamaged plant, artificial rolling and natural rolling plant treatments

Compound	Undamaged	Artificial rolling	Natural rolling
(a) after 12 h attack			
Hexanol, 2-ethyl-	3.39 ± 0.19 b	4.42 ± 1.19 b	12.89 ± 1.33 a
β-Linalool	6.64 ± 2.2	4..6 ± 0.73	7.69 ± 2
Nonanal	4.98 ± 0.2 b	5.07 ± 0.99 b	13.71 ± 1.33 a
Camphor	0.58 ± 0.06	2.33 ± 1.01	1.12 ± 0.17
Decanal	5.78 ± 0.69 b	5.64 ± 1.23 b	21.18 ± 1.65 a
Ethylacetophenone	0.59 ± 0.04 b	1.29 ± 0.24 a	1.39 ± 0.08 a
Methyl salicylate	0.42 ± 0.03	1.08 ± 0.54	0.54 ± 0.18
D-Limonene	1.2 ± 0.12	1.88 ± 0.58	2.25 ± 0.42
Decane, 3-methyl-	0.83 ± 0.25 b	1.59 ± 0.3 b	4.08 ± 1.13 a
Methyldecahydronaphthalene	0.35 ± 0.1	0.66 ± 0.16	1.62 ± 0.58
Undecane, 2-methyl-	2.86 ± 0.61	3.42 ± 0.95	6 ± 1.72
Undecane, 3-methyl-	5.91 ± 0.46 b	5.23 ± 0.77 b	12.52 ± 1.84 a
Dodecane	2.62 ± 0.47	4.75 ± 2.01	5.07 ± 1
Tetradecane	9.12 ± 0.64	12.55 ± 2.97	12.73 ± 0.67
α-Cedrene	4.31 ± 0.3	5.66 ± 1.75	5.9 ± 0.2
Pentadecane	8.69 ± 0.61	18.01 ± 5.86	13.81 ± 0.89
Pentadecane, 2-methyl-	1.46 ± 0.13	2.42 ± 0.69	2.3 ± 0.25
Hexadecane	8.75 ± 0.6	12.34 ± 4.14	12.66 ± 0.91
Pentadecane, 2,6,10-trimethyl-	3.69 ± 0.23	4.31 ± 1.56	5.52 ± 0.59
Heptadecane	5.91 ± 0.5	5.64 ± 1.66	8.35 ± 0.51
Pentadecane, 2,6,10,14-tetramethyl-	3.58 ± 0.31	3.76 ± 0.9	4.61 ± 0.4
Tridecane, 3-methyl-	0.73 ± 0.06	1.63 ± 0.62	1.41 ± 0.2
(b) after 24 h attack			
Hexanol, 2-ethyl-	3.39 ± 0.19 b	3.58 ± 0.59 b	10.02 ± 0.85 a
β-Linalool	6.64 ± 2.2	23.04 ± 16.27	18.02 ± 5.77
Nonanal	4.98 ± 0.2 b	3.84 ± 0.72 b	10.61 ± 0.67 a
Camphor	0.58 ± 0.06 b	0.4 ± 0.12 b	1.03 ± 0.08 a
Decanal	5.78 ± 0.69 b	4.5 ± 0.91 b	12.77 ± 2.01 a
Ethylacetophenone	0.59 ± 0.04 b	0.89 ± 0.13 b	1.44 ± 0.16 a
Methyl salicylate	0.42 ± 0.03	0.27 ± 0.09	0.54 ± 0.11
D-Limonene	1.2 ± 0.12	3.34 ± 1.38	4.24 ± 1.03
Decane, 3-methyl-	0.83 ± 0.25	1.22 ± 0.33	2.74 ± 1.41
Methyldecahydronaphthalene	0.35 ± 0.1	0.27 ± 0.05	0.51 ± 0.19
Undecane, 2-methyl-	2.86 ± 0.61	1.1 ± 0.2	3.9 ± 1.51
Undecane, 3-methyl-	5.91 ± 0.46 ab	2.87 ± 0.74 b	8.04 ± 1.77 a
Dodecane	2.62 ± 0.47	3.44 ± 1.07	4.68 ± 0.65
Tetradecane	9.12 ± 0.64	9.99 ± 1.85	13.44 ± 0.65
α-Cedrene	4.31 ± 0.3	4.28 ± 0.56	6.34 ± 0.43
Pentadecane	8.69 ± 0.61	8.52 ± 1.49	13.09 ± 1.01
Pentadecane, 2-methyl-	1.46 ± 0.13	1.55 ± 0.39	2.14 ± 0.03
Hexadecane	8.75 ± 0.6	8.23 ± 1.36	12.6 ± 1.09
Pentadecane, 2,6,10-trimethyl-	3.69 ± 0.23	3.98 ± 0.98	5.65 ± 0.44
Heptadecane	5.91 ± 0.5	5.98 ± 1.21	8.82 ± 0.56
Pentadecane, 2,6,10,14-tetramethyl-	3.58 ± 0.31	3.55 ± 0.85	5.19 ± 0.36
Tridecane, 3-methyl-	0.73 ± 0.06	0.77 ± 0.11	1.21 ± 0.24

Note: Means marked with different letter indicates significant differences in compounds across treatments (Tukey’s post hoc test, *P* < 0.05)

### Global transcriptome changes in rice leaves in response to *Cnaphalocrocis medinalis* infestation

**Fig. 3:**
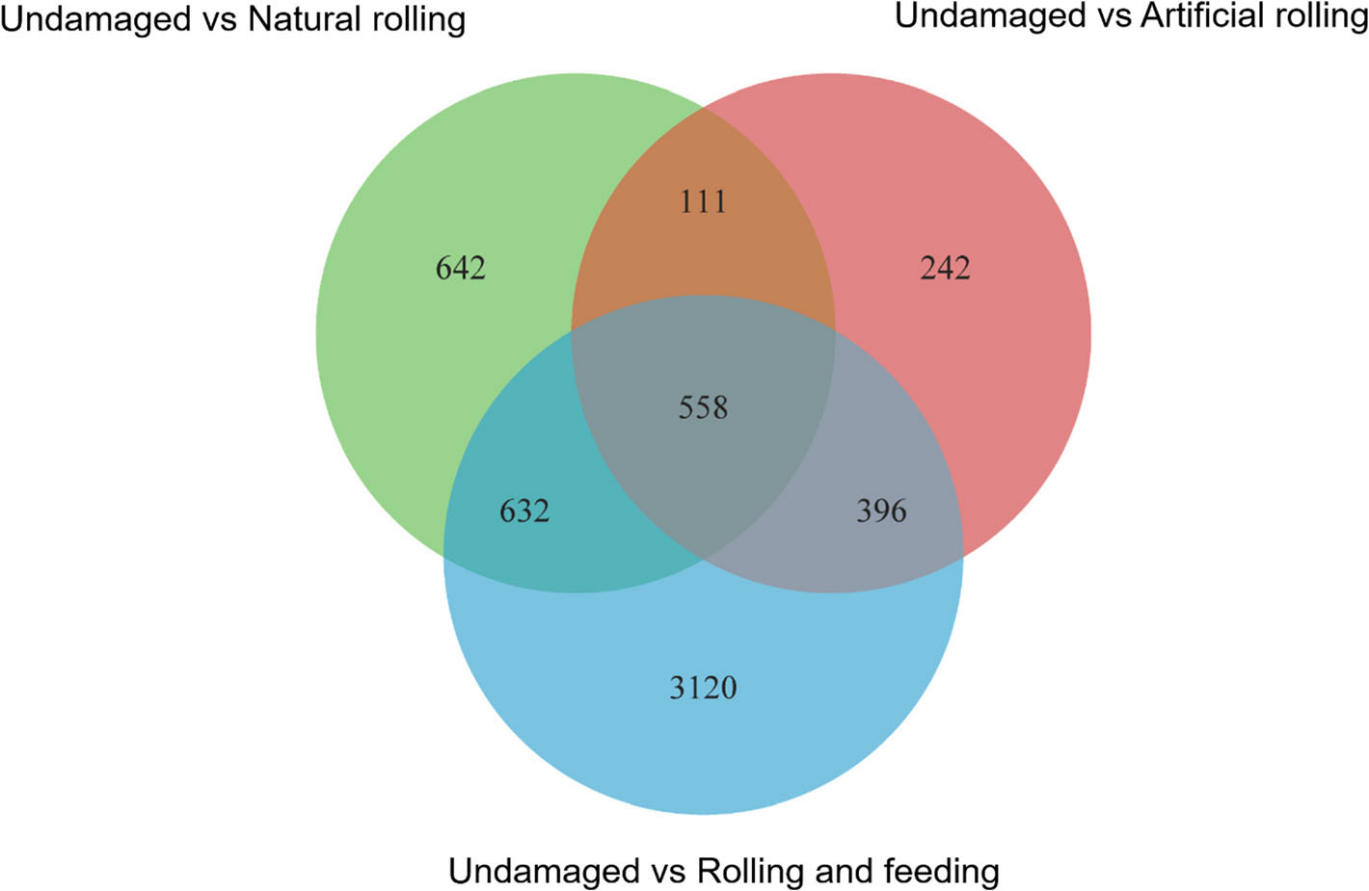
Comparative analysis of changes in the rice leaf transcriptome in response to artificial rolling, natural rolling and rolling and feeding by *Cnaphalocrocis medinalis* after 12 h. Genes associated with a *q* ≤ 0.05 for at least one time point were used to construct the Venn diagram

**Fig. 4:**
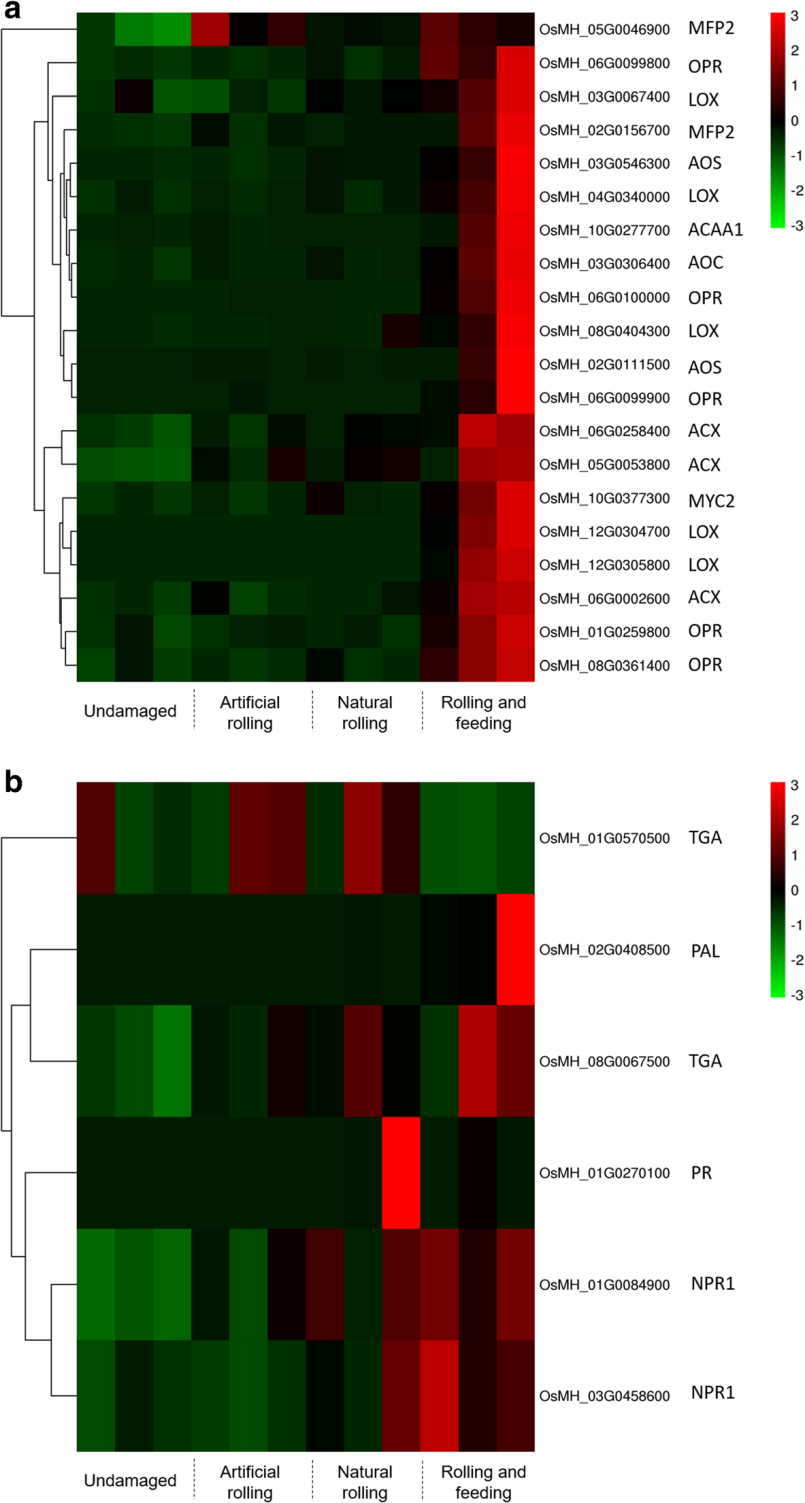
Comparative analysis of gene transcriptomes associated with jasmonic acid and salicylic acid pathways. **a** gene transcriptional differences related to jasmonic acid pathway and (**b**) gene transcriptional differences related to salicylic acid pathway. Note: enoyl-CoA hydratase/3-hydroxyacyl-CoA dehydrogenase (*MFP*2); 12-oxophytodienoic acid reductase (*OPR*); lipoxygenase (*LOX*); hydroperoxide dehydratase (*AOS*); acetyl-CoA acyltransferase 1 (*ACAA1*); allene oxide cyclase (*AOC*); transcription factor MYC2 (*MYC*2); acyl-CoA oxidase (*ACX*); *TGA* transcription factors (*TGA*); phenylalanine ammonia-lyase (*PAL*); pathogenesis-related (*PR*) protein; regulatory protein NPR1 (*NPR*1)

We used high-throughput sequencing to elucidate global transcript abundance in rice, and then compared the expression profiles of artificial rolling, natural rolling and rolling and feeding plant against undamaged plant. Compared to the undamaged plant, rolling and feeding treatment induced a drastic change in rice plant transcriptional levels while artificial rolling induced slight changes (Fig. 3). Compared to undamaged plant, 1073 genes were up-regulated and 870 genes were down-regulated in natural rolling plant while 2899 and 1807 genes were up-regulated and down-regulated in rolling and feeding plant, respectively (Table 2). The KEGG enrichment analyses showed that many defense- or stress-related pathways were activated, including plant hormone pathways (see Additional file 1: Figure S1). Moreover, the transcriptional responses of rice plant’s JA and SA pathways associated with the different treatments used in this study were identified using the GO tool (Fig. 4). Among these, genes such as lipoxygenase (*LOX*)*,* hydroperoxide dehydratase (*AOS*)*,* allene oxide cyclase (*AOC*)*,* 12-oxophytodienoic acid reductase (*OPR*) and their homologs associated with the JA pathways were up-regulated, especially following the rolling and feeding treatment (Fig. 4a). For the SA pathway, TGA transcription factors (*TGA*)*,* pathogenesis-related (*PR*) protein and regulatory protein NPR1 (*NPR1*) genes were up-regulated in natural rolling treatment while phenylalanine ammonia-lyase (*PAL*), *TGA* and *NPR1* genes were up-regulated in rolling and feeding treatment when compared to the undamaged treatment (Fig. 4b).

**Table 2:** Numbers of genes up- or down-regulated, whose transcript levels increased after the indicated treatments (|log_2_ Fold Change| > 1, *P*-value< 0.05)

Control	Case	Up-regulated	Down-regulated	Total DEGs
Undamaged	Artificial rolling	759	548	1307
Undamaged	Natural rolling	1073	870	1943
Undamaged	Rolling and feeding	2899	1807	4706

Note: *DEGs* Differentially expressed genes

### Jasmonic acid and salicylic acid analyses

**Fig. 5:**
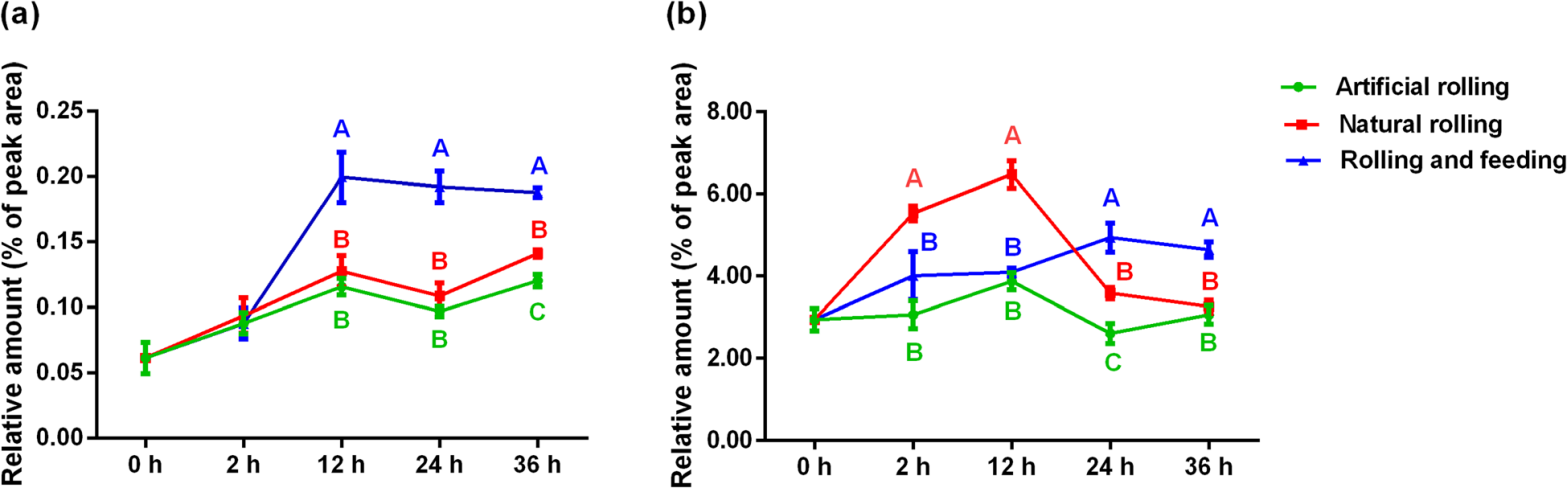
Plant phytohormones produced after natural rolling and rolling and feeding by *Cnaphalocrocis medinalis*, and artificial rolling of rice leaf at different time points. **a** jasmonic acid biosynthesis and (**b**) salicylic acid biosynthesis. Mean ± SEM. Means followed by different letter indicates significant differences between the treatments at specific time points (*P* < 0.01)

Based on transcript profile changes associated with hormone signaling, we measured JA and SA levels in natural rolling, rolling and feeding and artificial rolling treatments and compared to the undamaged plant by using LC/MS. Generally, similar and increasing trends in JA and SA biosynthesis levels were observed in both artificial rolling treatment and natural rolling treatment at all tested time points (Fig. 5). The accumulation of JA was greater in rolling and feeding treatment than other treatments from 12 h. Jasmonic acid level in natural rolling treatment showed no significant difference from 0 h to 24 h but was significantly higher when compared to its level in artificially rolled plant (*P <* 0.01, Fig. 5a). Salicylic acid level was significantly higher in natural rolling treatment after 2 h and 12 h than in other treatments (*P <* 0.01). However, rolling and feeding produced significantly higher levels of SA after 24 h and 36 h when compared to other treatments (*P <* 0.01, Fig. 5b).

## Discussion

**Fig. 6:**
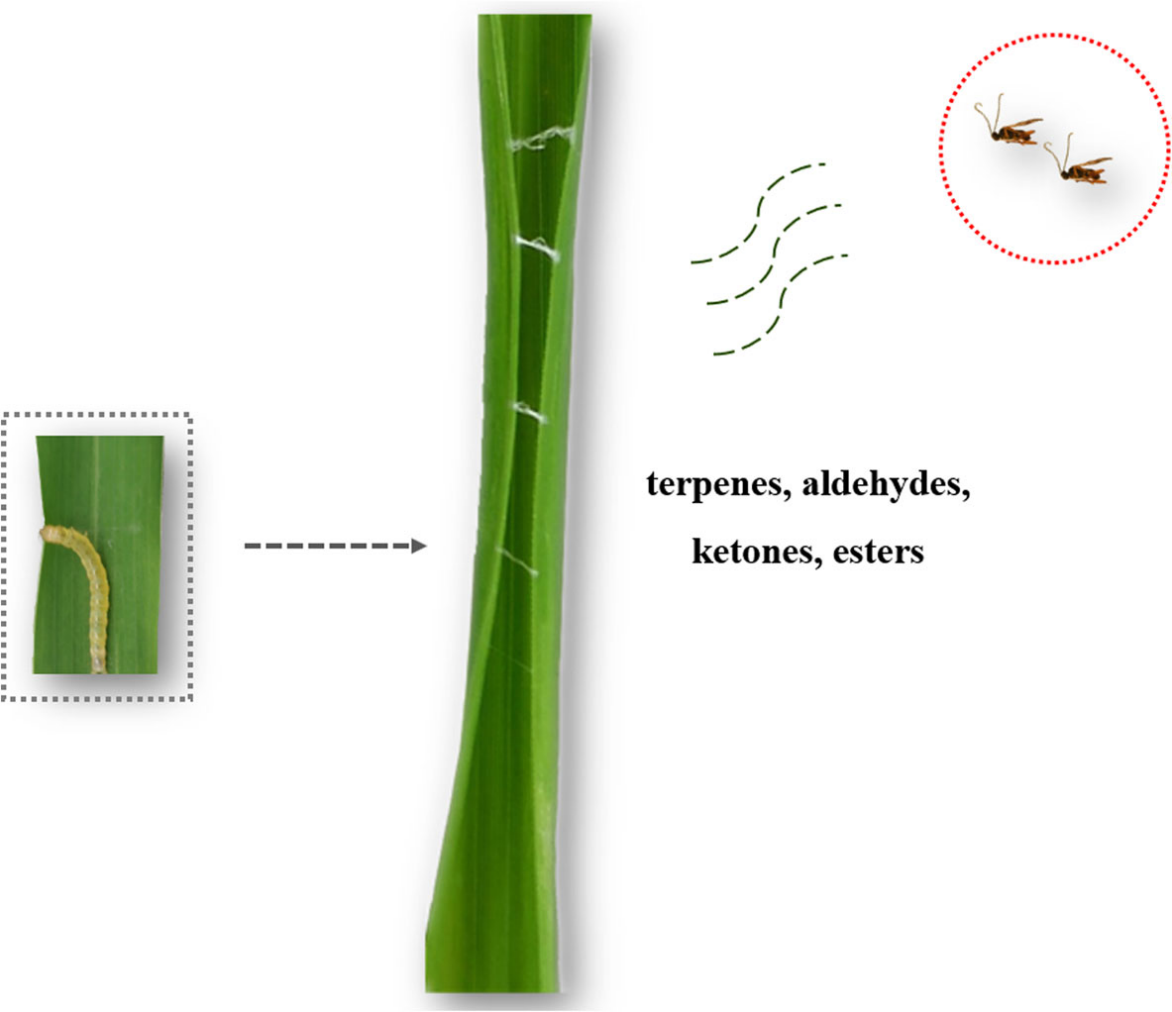
Rice plant can perceive the leaf rolling behavior of *Cnaphalocrocis medinalis*, release blends of volatiles (terpenes, aldehydes, ketones, esters) and recruit the natural enemy of the pest

The relationship between plants and insects has received much attention in recent years, with most studies focusing on plant resistance to herbivorous insects [43, 44]. Plant–insect co-evolution is essential for species diversity and survival [5]. For plants to develop effective and sustainable defenses against aggressors, early detection of the aggressors is essential [45]. In our study, rice leaf rolling by *C. medinalis* resulted in increased plant JA and SA levels, and in the release of plant volatile blends which helped the plant to attract natural enemies of the herbivore (Fig. 6).

Parasitoids often use different cues to locate their host [22]. In our study, we observed that *I. naranyae* significantly preferred naturally rolled plant over undamaged plant after 12 h of pest infestation. However, both wasp species showed no significant preferences for naturally rolled plant after 24 h induction when compared to undamaged plant. Similarly, both wasp species neither preferred undamaged nor artificially rolled plant after 12 and 24 h. There are no reports about the host cues employed by these two wasps in locating their target host. Thus, further studies should be carried out to unravel the cues involved in the wasps’ host location strategies.

Plant respond to stress in a series of steps after the stress is perceived [46, 47]. In general, wounding can induce direct and indirect plant defenses, such as changes in plant volatiles and attracting natural enemies of the attacker [48, 49]. Our results showed that natural rolling of rice leaf by *C. medinalis* significantly increased six volatile components while artificial rolling significantly increased only one volatile component after 12 h of induction. Principal component analysis showed that the headspace volatiles of natural rolling treated plant significantly differed, especially after 12 h of damage. We also found that the strong responses of wasp species to odors emanating from plants challenged by *C. medinalis,* was likely due to HIPVs, which modified their volatile organic compound (VOC) profiles when compared to undamaged plants.

Analysis of rice plant phytohormones showed significant differences in JA and SA levels in naturally rolled plant when compared to artificially rolled plant: for SA after 2 h to 24 h, and for JA after 36 h. It seems that leaf rolling behavior had a superimposed effect on plant immunity, when compared to artificial rolling. Therefore, our results suggest a correlation of plant defenses linked to *C*. *medinalis* leaf rolling behavior. The proteomics of *Bombyx mori* Linnaeus (Lepidoptera: Bombycidae), a silk-producing insect, revealed that many enzymes, protease inhibitors, and other unknown proteins are involved in plant defenses against its silk production. Among these, 40 protease inhibitors were identified as being involved in regulating or protecting the host plant from infestation [50]. However, the silk components from *C. medinalis*, which might have triggered plant responses, were not determined in the current study. Future studies should unravel these specific components and active ingredients involved in *C. medinalis* silk production and concomitant plant defenses. Additionally, treatments involving the application of silk only and application of silk and artificial rolling with silk maybe needed to assess whether similar defense responses of the plant will be recovered as reported in this study.

Our study also investigated plant defenses against feeding behaviors in order to explore differences in rice defense between rolling and feeding behaviors at the transcriptomic and metabolic level. Plant transcriptomic analysis showed an up-regulation of JA-related genes in the rolling and feeding treatment (Fig. 4a). Some SA-related genes were also up-regulated in the natural rolling and rolling and feeding treatments. Our result indicated that both natural rolling and rolling and feeding treatments could be perceived by rice plant, but the plant defense mechanisms differed in hormone levels. Jasmonic acid is famous for plant defense against insects’ attack and wounding. Clearly, feeding greatly increase the amount of JA when compared to JA levels in other treatments. Salicylic acid also plays an important role in early defense against herbivore insects. For instance, both *Helicoverpa zea (Boddie) (Lepidoptera: Noctuidae) and Pieris rapae (Linnaeus) (Lepidoptera: Pieridae) infestation raised SA level in cotton and arabidopsis, respectively* [14, 51]*.* We recommend further studies to determine whether JA–SA synergism could occur at the level of gene expression in *C. medinalis*-challenged MH63 rice plant. Further studies are also needed to determine the mechanisms, the elicitors, and the coupled gas chromatography/electroantennogram detection active components involved in this tritrophic system.

## Conclusions

Our study showed that similar to other tritrophic systems, HIPVs and the phytohormones JA and SA mediated rice-*C. medinalis*-*I. naranyae* tritrophic interactions [22, 52]. We provide the first evidence that rolling of rice leaves by *C. medinalis* induces JA and SA signaling pathways and HIPVs emission as plant defenses. In addition, our study showed differences in rice defenses among artificial rolling, natural rolling and rolling and feeding treatments. This could be due to the presence of different elicitors in rice’s ability to detect and defend itself against its aggressors. Our findings also provide a new beginning in exploring the effects of leaf rolling for silk-producing insect on associated plants and natural enemies.

## Methods

### Plants and insects

In this study, we used the rice (*Oryza sativa* L.) indica variety Minghui 63 (MH63), a restorer line with genetic stability. Single pre-germinated seeds of MH63 were sown in plastic pots (10 cm diameter × 8 cm in height), filled with a mixture of 0.5 g compound fertilizer (N:P:K = 14%:16%:15%, respectively) in a greenhouse at Huazhong Agricultural University, China. Plants were grown under natural light conditions at 28 ± 4 °C, 75 ± 5% relative humidity, and photoperiod ratios of 12/12 h, light/dark. The plants were watered daily and used at the tillering stage for both rearing and experimenting.

The *C. medinalis* colony was initiated from naturally occurring individuals, collected from rice fields at Xiaogan (113.91°E; 31.92°N), China. The insects were maintained in the laboratory on rice at 26 ± 2 °C, 75 ± 5% relative humidity, and photoperiod ratios of 16/8 h, light/dark. The fifth instar stage of the insect pest was used for all the experiments.

Adults of *I. naranyae* and *Apanteles* sp*.* were collected from paddy rice fields in Xiaogan, China and used in the Y-tube olfactomer experiment. Prior to the experiments, adults of each parasitoid species were fed on 10% honey solution until mating. After mating, parasitoids were transferred to the behavioral laboratory, at least 30 min prior to bioassay, to allow them to acclimatize to the experimental conditions.

### Plant treatments

Flag leaf of rice plant was subjected to the following treatments: (i) undamaged (healthy rice plant); (ii) artificial rolling; (iii) natural rolling (leaf rolling without feeding by *C. medinalis*); and (iv) rolling and feeding (*C. medinalis* rolled and fed leaf). For artificial rolling treatment, we used a white thin thread made of inorganic materials to tie around the rice leaf to mimic *C. medinalis* silk spinning and leaf rolling behavior. For natural rolling treatment, insects were carefully observed and removed from the leaf immediately after rolling was completed, to prevent feeding on the leaves. Individual plants were induced by artificial rolling and natural rolling for 12 or 24 h before olfactometer assays and volatile analysis. Plants were induced by artificial rolling, natural rolling and rolling and feeding for 12 h before transcriptomic analysis and induced by artificial rolling, natural rolling and rolling and feeding for 2, 12, 24, or 36 h before phytohormonal analysis. Healthy undamaged plants acted as the control.

### Behavioral response of parasitoid species to *Cnaphalocrocis medinalis* induced host plant volatiles

Responses of *I. naranyae* and *Apanteles* sp. females to different plant odor sources, in the absence of any visual cues, were tested using a Y-tube olfactometer (arm length: 18 cm and internal diameter: 1.5 cm). One arm of the tube was connected to the odor source and the other arm to the control treatment. A 60 W incandescent lamp bulb provided illumination. Charcoal-filtered clean air was passed through Teflon tubes into each arm of the olfactometer at a flow rate of 200 mL min^− 1^, and pulled out of the main arm of the olfactometer at the same rate, by a battery-powered portable vacuum pump (Sensen, Zhejiang, China).

Individual female parasitoid species were released one at a time in the main arm of the Y-tube. The pump system was turned on to test preferences for either the test or control odor, and each female was given 5 min to make a choice. The position of the test and the control arms of the Y-tube were changed after every three tested parasitoids to avoid positional bias. The connecting Teflon tubes and plant treatments were routinely replaced after six trials. A minimum number of 15 individual female parasitoid species acting as replicates were used for each pair of tests and control odor sources. The same environmental conditions, as described above, were maintained in the bioassay room.

### Collection and analysis of plant volatiles

We used a closed-loop dynamic headspace volatile collection system to collect volatiles for 8 h, as described by Sun et al. [53]. Purified air was pushed into a glass jar containing each plant treatment at 500 mL min^− 1^ and drawn from the jar through vacuum containing traps, packed with Super Q adsorbent (200 mg each; Alltech Associates Inc. Deerfield, IL, USA). The air purification system consisted of (1) charcoal (activated carbon, 6 to 14 mesh, Fisher Scientific), (2) 5A molecular sieves (beads, 8 to 12 mesh, Sigma-Fluka), and (3) silica gel Rubin (drying agent free of metal salts, silica gel, Sigma-Fluka). The collection was replicated four times for each odor source.

After 8 h of volatile trapping using Super Q, each filter was eluted with 1 mL of N-hexane acetate, spiked with 10 μL of internal standard (0.1 mg/mL nonyl acetate, Sigma), and stored at − 40 °C until chemical analysis. Volatile extracts were analyzed by coupled gas chromatography/mass spectrometry (GC/MS) (QP-2010, Shimadzu, Shiga, Japan), equipped with an HP-5 MS fused-silica column (30 m × 0.25 mm × 0.25 μm) (Agilent Technologies, USA). Helium (1 mL min^− 1^) was used as the carrier gas, and an initial oven temperature of 40 °C was maintained for 1 min, ramped at 8 °C min^− 1^ to 300 °C, and held for 5 min. Compounds were identified using the quasi-molecular ions. In addition, structural assignments of several compounds were confirmed using authentic standards on the GC/MS under the same conditions employed for crude volatile analysis. Quantification was based on calibration curves (peak areas) generated from authentic standards of identified compounds. The peak area of each component was compared to the percent relative amount of internal standard peak area.

### RNA isolation, library construction and sequencing

Total RNA extractions of plant leaves were performed using Trizol Reagent (Invitrogen Life Technologies). RNA purity and quantity were determined using a Nanodrop ND 1000 instrument (Nanodrop Technologies, Wilmington, DE, USA). Sequencing libraries were generated using the TruSeq RNA Sample Preparation Kit (Illumina, San Diego, CA, USA). Each treatment included three biological replicates.

### Bioinformatics analysis

Gene expression patterns were clustered using Cutadapt (v1.15) software to filter the sequencing data and to generate high quality sequences (clean data) for further analysis. Genes were mapped to ‘terms’ in the Gene Ontology (GO) database and the number of differentially enriched genes were calculated for each term. DESeq (1.30.0) was used to analyze differential gene expression with screening conditions as follows: expression difference multiple |log_2_FoldChange| > 1 and significance of *P* < 0.05. Then, terms with significant enrichment of differentially enriched genes were calculated by hypergeometric distribution. GO enrichment analysis was used to obtain GO functional terms with significant enrichment of differentially expressed genes and revealed the possible functions. Kyoto Encyclopedia of Genes and Genomes (KEGG) pathway enrichment analysis was used to conduct the enrichment analysis of differentially expressed genes. We counted the number of differentially expressed genes at different levels of KEGG pathway, and determined the metabolic pathways and signaling pathways that the differentially expressed genes mainly participated in. For each treatment, three biological replicates were used from individual plants.

### Analysis of jasmonic acid and salicylic acid

Rice leaves (approximately 50 mg fresh weight) were collected, immediately frozen in liquid nitrogen, and stored at − 80 °C until analysis. Analysis was performed as described previously [55]. After grinding in liquid nitrogen, the 50 mg of each sample was extracted in 500 μL extraction solvent (2-propanol/H_2_O/concentrated HCl, v/v/v) in a 2:1:0.002 ratio and spiked with 10 μL internal standards (5 μg/mL Dihydrojasmonic acid and 5 μg/mL D4-Salicylic acid; Sigma). Samples were homogenized in a thermostatic mixer (MTC-100, Guangzhou, China) for 30 min at 100 rpm and at 4 °C. Then, 1 mL dichloromethane was added to the samples and homogenized under the same conditions. Samples were centrifuged at 13,000 g and at 4 °C for 5 min, and the bottom aqueous phase collected into clean 2 mL Agilent bottles. Samples were then dried completely under a hooded chamber using nitrogen evaporator and dissolved by adding 100 μl of methanol. A 0.22 μm organic filtration membrane was used to purify the samples and stored at − 20 °C until analysis.

Samples were analyzed using a liquid chromatography/mass spectrometry (LC/MS) system (Xevo G2-XS Qtof) and maintained in a thermostat-controlled chamber at 4 °C. Jasmonic acid and SA levels were calculated from the ratio of the endogenous hormone peak and the known internal standard. The peak area of each component was compared to the percent relative amount of internal standard peak area. For each treatment, four to six replicates were sampled.

### Data analyses

Chi-squared tests were used to show differences in parasitoid species for the different categories of host plant odors. One-way ANOVA was used to show differences in phytohormone (JA and SA) levels and volatile components for the different treatment categories. Principal component analysis of plant volatiles was conducted and plotted using SIMCA software. High-throughput sequencing was used to elucidate global transcript abundance in rice plant, and compared the expression profiles of undamaged, artificial rolling, natural rolling and rolling and feeding plant treatments. All tests were performed at *P* < 0.05 statistical significance level and all data were analyzed in SPSS version 19.0 software.

## Supplementary information

**Additional file 1: Figure S1.** Kyoto Encyclopedia of Genes and Genomes of rice plants. (a) undamaged plant vs natural rolling plant by *Cnaphalocrocis medinalis* and; (b) artificially rolled plant vs natural rolling plant by *C. medinalis*, (c) natural rolling plant vs rolling and feeding by *C. medinalis*.

## Data Availability

The datasets used and/or analyzed during the current study are available from the corresponding author upon reasonable request.

## Abbreviations

GC/MS: Gas chromatography/mass spectrometry
HIPVs: Herbivore-induced plant volatiles
JA: Jasmonic acid
LC/MS: Liquid chromatography/mass spectrometry
MH63: Minghui 63
SA: Salicylic acid
VOC: Volatile organic compounds
