# Supplementary material for: Rice defense responses are induced upon leaf rolling by an insect herbivore
Source: BMC Plant Biol. 2019 Nov 25;19:514. doi: 10.1186/s12870-019-2116-0 (PMC6878700; doi:10.1186/s12870-019-2116-0)
Supplement: Supplementary file 1 — Additional file 1: Figure S1. Kyoto Encyclopedia of Genes and Genomes of rice plants. (a) undamaged plant vs natural rolling plant by Cnaphalocrocis medinalis and; (b) artificially rolled plant vs natural rolling plant by C. medinalis, (c) natural rolling plant vs rolling and feeding by C. medinalis. [file 12870_2019_2116_MOESM1_ESM.docx]

**Additional file 1**


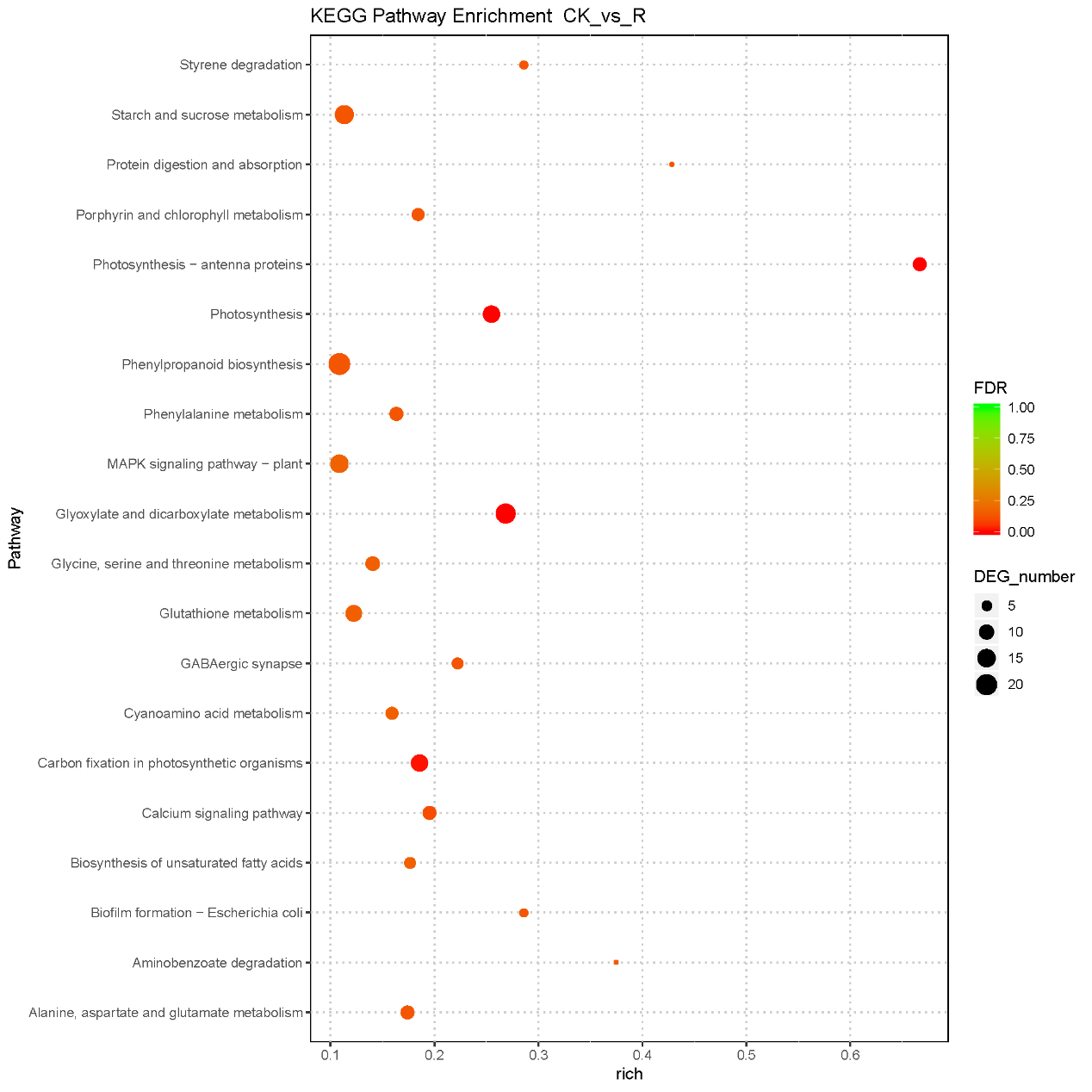


(a) CK vs R


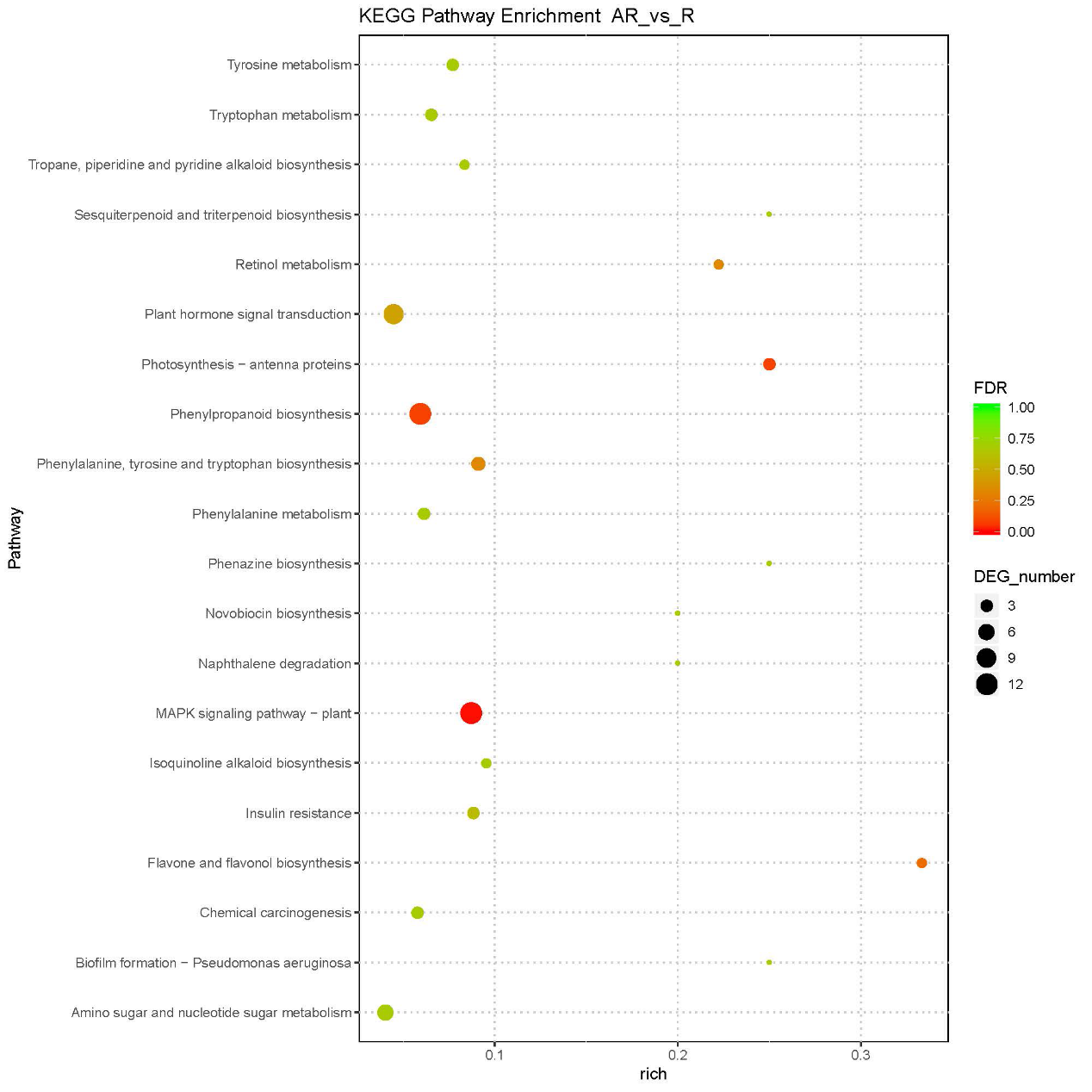


(b) AR vs R


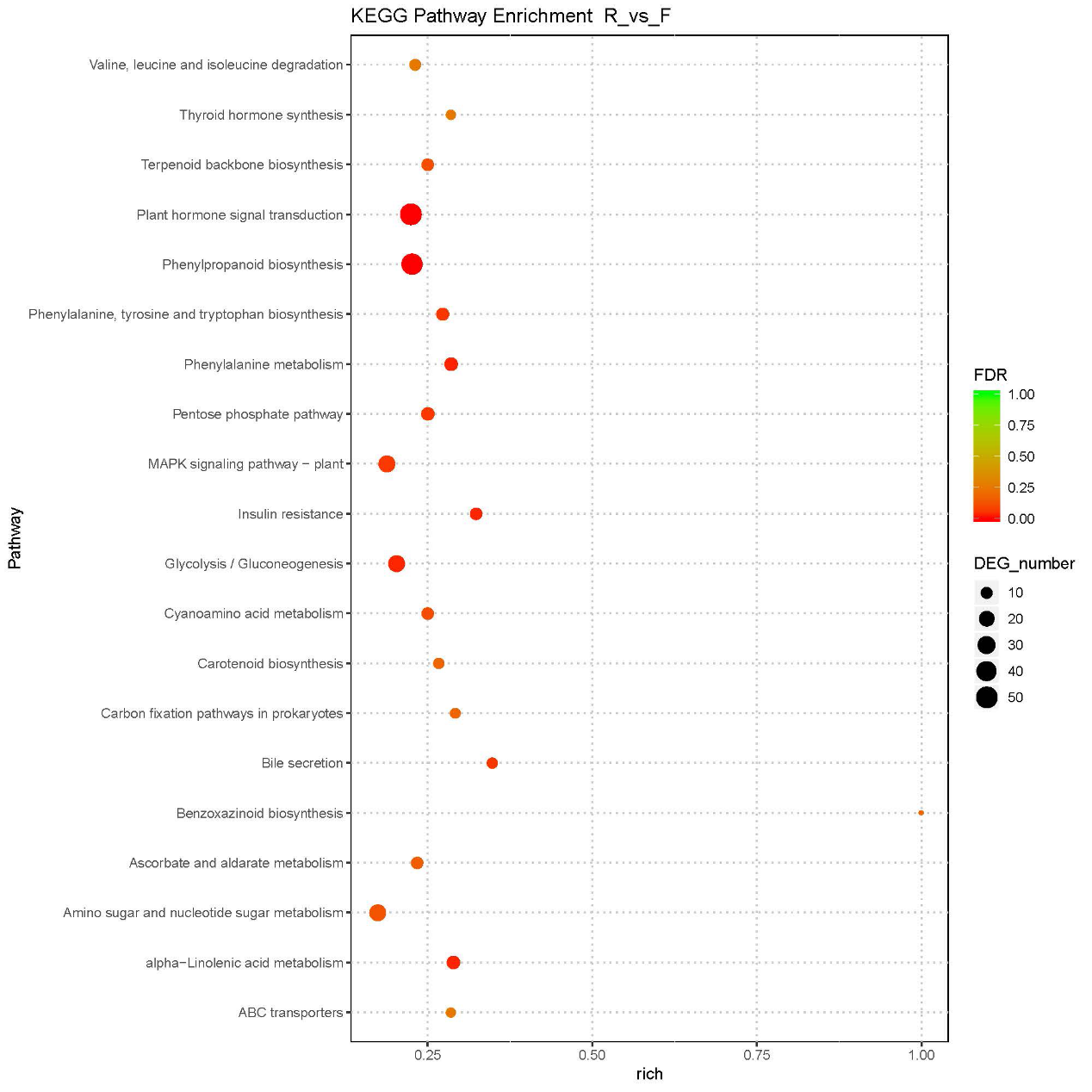


(c) R vs F

**Figure S1. KEGG enrichment analysis of natural-rolling (R) and natural rolling and feeding (F) by *Cnaphalocrocis medinalis*, undamaged plants (CK) and artificial-rolling (AR).** (a) CK vs R; (b) AR vs R; (c) R vs F. The size of the dots in the figure represents the ratio of the number of differentially expressed genes enriched in the pathway to the number of all genes annotated in the pathway, The color represents the significance P value of the pathway.
